# Dendritic Cells from Crohn’s Disease Patients Show Aberrant STAT1 and STAT3 Signaling

**DOI:** 10.1371/journal.pone.0070738

**Published:** 2013-08-07

**Authors:** Janne K. Nieminen, Mirja Niemi, Taina Sipponen, Harri M. Salo, Paula Klemetti, Martti Färkkilä, Jukka Vakkila, Outi Vaarala

**Affiliations:** 1 Immune Response Unit, Department of Vaccination and Immune Protection, National Institute for Health and Welfare, Helsinki, Finland; 2 Department of Medicine, Division of Gastroenterology, Helsinki University Central Hospital, Helsinki, Finland; 3 Hematology Research Unit, University of Helsinki and Helsinki University Central Hospital, Helsinki, Finland; INSERM, France

## Abstract

Abnormalities of dendritic cells (DCs) and STAT proteins have been reported in Crohn’s disease (CD). Studies on JAK/STAT signaling in DCs are, however, lacking in CD. We applied a flowcytometric single-cell-based phosphoepitope assay to evaluate STAT1 and STAT3 pathways in DC subsets from CD patients. In addition, circulating DC counts were determined, together with the activation-related immunophenotype. We found that IL-6- and IFN-α-induced STAT3 phosphorylation and IFN-α-induced STAT1 phosphorylation were impaired in plasmacytoid DCs (pDCs) from CD patients (*P* = 0.005, *P = *0.013, and *P = *0.006, respectively). In myeloid DCs (mDCs), IFN-α-induced STAT1 and STAT3 phosphorylation were attenuated (*P*<0.001 and *P = *0.048, respectively), but IL-10-induced STAT3 phosphorylation was enhanced (*P* = 0.026). IFN-γ-induced STAT1 signaling was intact in both DC subtypes. Elevated plasma IL-6 levels were detected in CD (*P* = 0.004), which strongly correlated with disease activity (ρ = 0.690, *P*<0.001) but not with IL-6-induced STAT3 phosphorylation. The numbers of pDCs and BDCA3+ mDCs were decreased, and CD40 expression on CD1c+ mDCs was increased in CD. When elucidating the effect of IL-6 signaling on pDC function, we observed that IL-6 treatment of healthy donor pDCs affected the maturation of and modified the T-cell priming by pDCs, favoring Th2 over Th1 type of response and the expression of IL-10 in T cells. Our results implicate DC signaling in human CD. Reduced IL-6 responsiveness in pDCs, together with the attenuated IFN-α-induced signaling in both DC subtypes, may contribute to the immunological dysregulation in CD patients.

## Introduction

Dendritic cells (DCs) are professional antigen-presenting cells capable of priming and activating T cells. Since an abnormal T-cell activation is an inherent feature of Crohn’s disease (CD) and is also seen in several models of intestinal inflammation [1], aberrant DC function may contribute to the disease process. Various DC subsets have been identified in humans [2], [3], of which myeloid (mDCs) and plasmacytoid DCs (pDCs) constitute the main categories. mDCs represent the conventional DC subtype, which, after receiving maturation stimuli through pathogen-recognition receptors, activate T cells for immune responses. On the other hand, immature or semimature mDCs can induce T-cell anergy or tolerance [4], [5]. pDCs are also efficient in activating T cells for immune responses under certain circumstances, such as in the context of antiviral immunity [6], [7], but an increasing body of evidence also suggests a role for pDCs in the induction and maintenance of T-cell mediated immune tolerance [8]–[13].

In CD, DCs have been investigated to some extent, although functional studies on human DCs are currently scarce. Alterations in blood, intestinal or mesenterial lymph node (MLN) DC numbers have been reported, together with changes in maturation marker expression [14]–[20].

DCs receive via cytokines guidance in accommodating the varying requirements of homeostasis and infection appropriately. The signals from several cytokines are delivered intracellularly by the JAK-STAT pathway. After the ligation of the cytokine receptor on the cell surface, Janus kinases (JAK) are recruited to phosphorylate Signal Transducers and Activators of Transcription (STAT) family of proteins with a certain specificity. STAT proteins then dimerize, translocate to the nucleus, and function as transcription factors. In CD, alterations in the expression and phosphorylation of STAT proteins have been reported [21]–[26], especially those involving STAT3, but no studies have focused on DCs. The role of STAT3 signaling in the pathogenesis is also implicated by the association of a *STAT3* polymorphism with CD [27]. STAT3 activity is considered necessary to keep DC maturation under control [28], [29], whereas STAT1 signaling is indispensable for normal maturation [30].

To assess whether cytokine-induced JAK-STAT signaling in DCs is altered in CD patients, we further developed flow cytometry-based methodology [31] to enable single-cell level examination of STAT1 and STAT3 phosphorylation in blood-derived mDCs and pDCs. We found impaired interleukin (IL)-6- and interferon (IFN)-α- but not IL-10-induced signaling in pDCs of CD patients. In mDCs, IFN-α-induced signaling was similarly attenuated, while IL-10-induced STAT3 phosphorylation was enhanced. The functional *in vitro* experiments revealed that IL-6-treated pDCs promoted IL-4 and IL-10 expression in co-cultured T cells, which suggests that the impaired IL-6/STAT3 signaling in pDCs may affect T-cell regulation.

## Materials and Methods

### Patients and Control Subjects

A total of 22 patients with CD (mean age 40.4, 13 males; detailed characteristics are specified in Table 1) came to the clinic for endoscopic follow-up and were consequently enrolled for DC enumeration, surface marker expression and STAT phosphorylation studies. The control group (mean age 37.4, eight males) comprised 21 volunteers without autoimmune diseases or acute infection. The expression of selected cytokines in isolated pDCs was studied in a separate series of patients (n = 11, mean age 28.1, six males) and control subjects (n = 11, mean age 31.6, five males). One patient and two controls were common to both series at separate time points. *In vitro* function of pDCs was studied using blood samples from healthy volunteers. T cells were obtained from general population blood donor buffy coats (Finnish Red Cross). All participants gave written informed consent, and the study was approved by the ethics committee of the Helsinki University Central Hospital. Research was conducted in accordance with the Declaration of Helsinki.

**Table 1:** Patient characteristics in DC enumeration, surface marker expression and STAT phosphorylation studies.

Age	Sex	Time with CD (y)	Pattern	Localization	Medication for CD	CDAI	SES-CD	ESR	CRP	Analysis of phosphorylated STATs; stimulation with	Analysis of DC counts & surface markers
20	m	8	inflammatory+PD	colon	AZA, prednisone, IFX	25	14	N/A	<3	IFN-α, IFN-γ, IL-6, IL-10	+
41	m	8	inflammatory	ileocolon	AZA, 5-ASA, prednisone, IFX	251	16	N/A	19	IFN-α, IFN-γ, IL-6, IL-10	+
40	m	16	stricturing	colon	AZA, 5-ASA	45	13	12	7	IFN-α, IFN-γ, IL-6, IL-10	+
23	m	8	stricturing	ileocolon	AZA, 5-ASA	43	12	18	<3	IFN-α, IFN-γ, IL-6, IL-10	+
42	f	N/A	penetrating	ileum	none	33	5	N/A	<3	IFN-α, IFN-γ, IL-6, IL-10	+
56	f	11	stricturing	ileocolon	5-ASA	141	9	N/A	5	IFN-α, IFN-γ, IL-6, IL-10	+
38	f	23	penetrating+PD	ileocolon, upper GI	5-ASA, MTX	98	14	N/A	23	IFN-α, IFN-γ, IL-6, IL-10	+
52	f	9	inflammatory	colon	5-ASA, 6-MP	44	0	16	12	IFN-α, IFN-γ, IL-6, IL-10	−
51	f	38	penetrating	ileocolon	none	28	8	N/A	5	IFN-α, IFN-γ, IL-6, IL-10	−
39	m	20	stricturing	ileocolon, upper GI	AZA	28	0	2	<3	IFN-α, IFN-γ, IL-6, IL-10	−
60	m	4	inflammatory	colon	5-ASA	48	0	11	<3	IFN-α, IFN-γ, IL-6, IL-10	−
63	m	29	stricturing	ileum	AZA	75	4	7	<3	IFN-α, IFN-γ, IL-6, IL-10	+
50	m	18	stricturing	ileum	none	154	7	19	11	IFN-α, IFN-γ, IL-6	+
44	m	21	stricturing	ileum	AZA, 5-ASA	103	0	N/A	N/A	IFN-α, IFN-γ, IL-6	+
40	m	7	penetrating+PD	ileum	AZA, 5-ASA	98	9	<2	<3	IFN-α, IFN-γ, IL-10	+
22	m	1.2	inflammatory	ileocolon, upper GI	AZA, 5-ASA, IFX	88	12	3	6	IFN-α, IFN-γ	+
35	f	5	stricturing	ileocolon	AZA, 5-ASA	99	26	28	26	IFN-α, IFN-γ	+
23	f	10	inflammatory+PD	ileocolon	AZA, 5-ASA, prednisone	158	32	63	54	IFN-α, IFN-γ	+
20	m	0.4	inflammatory+PD	ileocolon	5-ASA, prednisolone	183	21	8	<5	−	+
38	m	16	stricturing	ileum	AZA, 5-ASA, budesonide	298	13	6	<5	−	+
40	f	28	stricturing+PD	ileocolon	AZA	149	8	21	<3	−	+
39	f	14	stricturing+PD	ileum	AZA, sulphasalazine	83	6	N/A	N/A	−	+

Abbreviations: PD = perianal disease; AZA = azathioprine; IFX = infliximab; 5-ASA = 5-aminosalicylic acid; MTX = methotrexate; 6-MP = 6-mercaptopurine; ESR = erythrocyte sedimentation rate, mm/h; CPR = C-reactive protein, mg/L; CDAI = Crohn's disease activity index, generally interpreted as follows: <150 = inactive disease, ≥150 = active disease; SES-CD = Simple Endoscopic Score for Crohn's disease, generally interpreted as: ≤3 = inactive disease, 4–14 = mild to moderate disease, ≥15 = severe disease.

### Evaluation of Endoscopic Disease Activity

Colonoscopies were performed by experienced gastroenterologists who evaluated the disease activity using the Simple Endoscopic Score for Crohn’s Disease (SES-CD) [32]. Venous blood samples for DC analyses were taken within three days of colonoscopy (except in three cases within ten days).

### Staining of Cells for Phenotyping and Counting

200 µl of heparinized blood was added to monoclonal antibodies (mAbs, specified in Table S1) for 20 minutes, and erythrocytes were then lysed with FACS Lysing Solution (BD Biosciences, San Jose, CA). After two washes with washing buffer consisting of 5% fetal bovine serum (FBS) and 0.02% (w/v) sodium azide in phosphate-buffered saline (PBS), cells were suspended in 1% (w/v) paraformaldehyde (PFA) in PBS and stored overnight at 4°C. For isolated and cultured cells, the lysing step and PFA were omitted, and cells were stained and analyzed in washing buffer on the same day. 7-amino-actinomycin D (7-AAD, eBioscience, San Diego, CA) was used with isolated pDCs to exclude dead cells.

### Cytokine Stimulation and Staining of Cells for Measurement of Phosphorylated STATs

250 µl of heparinized blood was preincubated for 30 minutes at 37°C in a CO_2_ incubator, and pre-mixed cocktails of mAbs against cell-surface markers (specified in Table S1) were quickly pipetted to the tubes to enable DC identification. After additional 10 minutes at 37°C in a CO_2_ incubator, the following recombinant human cytokines were added: 1000 IU/ml IFN-α-2a, (Ropheron-A, Roche), 1000 IU/ml IFN-γ (Peprotech, Rocky Hill, NJ), 250 ng/ml IL-10 (Peprotech) or 50 ng/ml IL-6 (Peprotech). Samples were stimulated for 10 minutes at 37°C in a CO_2_ incubator, and 2 ml of FACS Lysing Solution was then added for 10 minutes at room temperature. The fixation was completed by adding 2 ml 3% (w/v) PFA in PBS for additional 10 minutes, and cells were then permeabilized with −20°C methanol at a final concentration of 80% (v/v) for 10 minutes on ice (no washing steps up to this point). Cells were spun down, washed twice with washing buffer (see above) now containing 10% FBS, and mAbs recognizing the tyrosine-phosphorylated forms of STAT1 (pY701; pSTAT1) or STAT3 (pY705; pSTAT3) molecules, or an isotype control mAb, were added for 30 minutes in washing buffer (10% FBS). Cells were then washed twice (5% FBS in the washing buffer), suspended in 1% PFA in PBS, and stored overnight at 4°C. STAT3 phosphorylation studies using peripheral blood mononuclear cells (PBMCs) isolated by density-gradient centrifugation were conducted in the same manner in RPMI 1640 culture medium (Gibco/Invitrogen, Carlsbad, CA) +2 mM L-glutamin, omitting the erythrocyte-lysing step.

### Flow Cytometry

**Figure 1:**
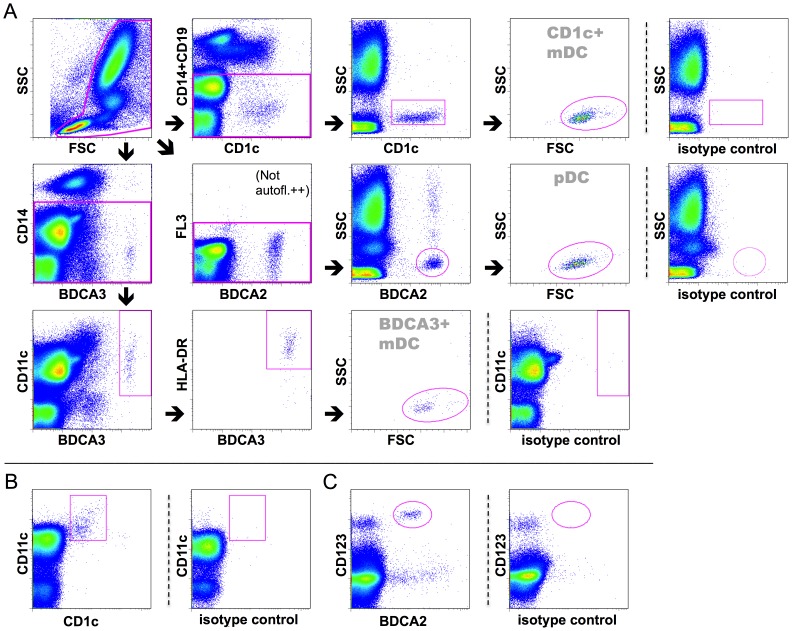
Gating strategy for flow cytometry. DC subtypes were identified for cell surface molecule analyses as shown. An example of the leukocyte gating first applied for all DC subsets is shown in the upper left corner (A). In pSTAT measurements, CD11c and CD123 were utilized as additional markers for CD1c+ mDCs (B) and pDCs (C), respectively; otherwise, a gating strategy similar to the cell surface marker protocol was used. A representative example of a healthy control is shown.

Samples were analyzed with FACSCalibur flow cytometer (BD Biosciences). Gating strategy for DC analyses is shown in Fig. 1. For the determination of pDC numbers, BDCA2+CD123 double staining was used for gating. Cell doublets were excluded based on light scatter, except for DC enumeration. The absolute DC numbers were calculated by multiplying the relative DC counts by the absolute leukocyte count (determined in a routine hematology laboratory). Cell surface molecule expression was quantified as median fluorescence intensity (MFI) in arbitrary units (AU) with isotype control intensity subtracted. The magnitude of pSTAT1 and pSTAT3 responses following stimulation was calculated as a fold increase over the unstimulated sample. FlowJo software (Tree Star, Inc., Ashland, OR) was used to analyze flow cytometry data.

### Isolation and Culture of pDCs

For cell-culture studies, PBMCs were obtained by density-gradient centrifugation, and non-pDCs were magnetically depleted using Plasmacytoid Dendritic Cell Isolation Kit and LS columns (Miltenyi Biotec, Gladbach, Germany), according to the manufacturer’s instructions. Median purity of the pDC fraction was 93.8%, as determined by flow cytometry (data not shown). pDCs were washed and plated at a density of 1.25×10^5^ cells/ml on U-bottom 96-well plates (Nunc, Roskilde, Denmark) in culture medium consisting of RPMI 1640, supplemented with 10% FBS, 2 mM L-glutamine, 50 µM 2-mercaptoethanol, antibiotics, and 10 ng/ml of recombinant human IL-3 (R&D Systems, Minneapolis, MN). The concentration of recombinant human IL-6 (Peprotech) was 50 ng/ml and type B (ODN 2006) CpG (InvivoGen, San Diego, CA) were used at 2 µM. pDCs were cultured at 37°C in a CO_2_ incubator for two days and harvested for flowcytometric analysis or for T-cell stimulation experiments after careful washing and counting of viable cells with Trypan Blue exclusion. Alternatively, pDCs were harvested for quantitative real-time PCR (RT-qPCR) analysis after 16 h of incubation. To obtain highly purified cells (>99%) for mRNA expression studies, pDCs were isolated from frozen and thawed PBMC fractions by combined depletion of non-pDCs and positive selection of BDCA4+ cells using Diamond Plasmacytoid Dendritic Cell Isolation Kit II with LD and MS columns (Miltenyi Biotec).

### Stimulation of Naive T Cells

PBMCs were isolated by density-gradient centrifugation, and naive T cells were negatively enriched using Naive CD4+ T Cell Isolation Kit II (Miltenyi Biotec) and LS columns, according to the manufacturer’s instructions. The purity of the CD4^+^CD45RA^+^ fraction thus obtained was routinely 96% or higher, as assessed by flow cytometry (data not shown). Cells were then stimulated with allogeneic pDCs as previously described [5], with some modifications. Briefly, naive T cells (20 000 cells per well in 200 µl) were activated for six days with the 2d cultured pDCs at a pDC-T cell ratio of 1∶5 in culture medium without IL-3 on U-bottom 96-well plates. Cells were harvested, washed, and viable cells were counted. At d6, T-cell proliferation was readily detectable by light microscopy. Because viable cells at d6 of co-culture consisted mainly of T cells, as assessed by flow cytometry (Fig. S1), no pDC depletion step prior to T-cell restimulation was included in the protocol. T cells were restimulated at a density of 5×10^5^ cells/ml on flat-bottom 96-well plates with plate-bound anti-CD3 (wells were coated with 5 µg/ml of anti-CD3) and 1 µg/ml of soluble anti-CD28 mAb (clones UCHT1 and CD28.2, respectively, both from BD Biosciences) for 16 hours, and supernatants were then collected and cells were lysed for RT-qPCR analyses.

### Extraction of RNA and RT-qPCR Analyses

Extraction of total RNA with RNeasy Micro Kit (Qiagen, Hilden, Germany) and RT-qPCR using Stepone plus real-time PCR systems sequence detector (Applied Biosystems, Foster City, CA) were performed as previously described [33]. The following gene products were measured: IFN-γ (Applied Biosystems’ assay Hs00174143_m1), IL-4 (Hs00174122_m1), IL-10 (Hs00961622_m1), FOXP3 (Hs00203958_m1), ICOS-L (Hs00323621_m1), PD-L1 (Hs01125299_m1), IDO (Hs00984151_m1), IL-6 (Hs00174131_m1), and IFN-α2 (Hs00265051_s1). Ribosomal 18S RNA (Hs99999901_s1 or Hs03928985_g1) was used as an endogenous control. Target gene expression was analyzed by the 2^−ΔΔCt^ method. For ratios, ΔCt between the target mRNAs was used (2^−ΔCt^).

### Measurements from Plasma and Culture Supernatant Samples

FlowCytomix Multiplex Technology kits (Bender MedSystems, Vienna, Austria, and eBioscience) were used according to the manufacturer’s instructions to measure cytokine concentrations. Data was acquired with FACSCalibur flow cytometer and analyzed using FlowCytomixPro software (v2.3, eBioscience) and Prism 5 software (GraphPad Software Inc., La Jolla, CA). Concentrations of the DC growth factor FLT3 ligand in plasma samples were determined using a component from Milliplex® MAG Human cytokine/chemokine kit (HFLT3L-MAG, Millipore Corporation, Billerica, MA) according to the manufacturer’s instructions, and the analysis was done with Magpix system and xPonent software (Luminex Corporation, Austin, TX), and with Prism 5 software.

### Statistical Analyses

Statistical analyses were performed using PASW Statistics (v18.0, SPSS Inc., Chicago, IL) or Prism 5 software. Unpaired t-test was used for comparisons between two groups when appropriate; otherwise, Mann-Whitney U test was used. Multiple groups were compared with Kruskal-Wallis test. Dichotomous data were analyzed with Fisher’s exact test. In cell culture experiments, paired t-test or Wilcoxon matched pairs test was used to compare the effects of IL-6 treatment. Spearman’s rank correlation was utilized to examine the correlation between two parameters. A *P* value <0.05 was considered statistically significant.

## Results

### CD Patients have Decreased Numbers of Circulating pDCs and BDCA3+ mDCs

**Figure 2:**
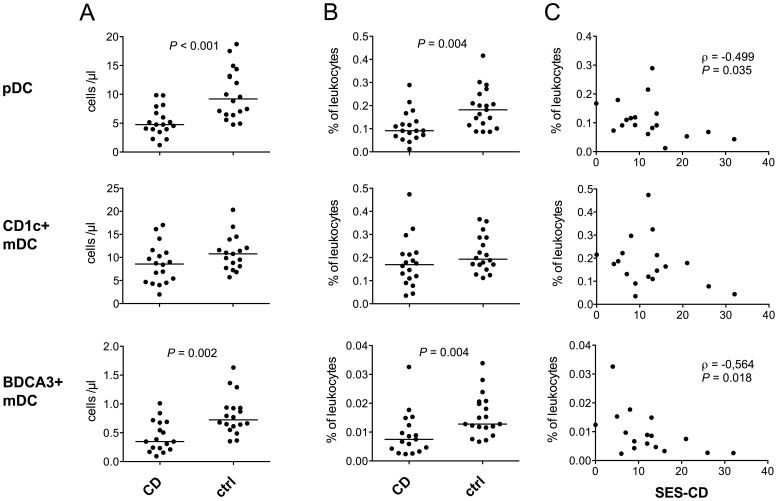
Patients with Crohn’s disease have reduced numbers of pDCs and BDCA3+ mDCs in the peripheral blood. Both absolute (A) and relative (B) pDC and BDCA3+ mDC counts are decreased in CD. The major blood myeloid DC population expressing CD1c is not significantly different from controls in size. Horizontal lines indicate median levels. Statistically significant negative correlations between the total SES-CD score and relative pDC and BDCA3+ mDC counts are observed in CD patients (C).

Both absolute and relative numbers of pDCs were decreased in CD (median 4.76 vs. 9.93 cells/µl, *P*<0.001; 0.0915 vs. 0.182% of leukocytes, *P* = 0.004), as demonstrated in Fig. 2A and 2B. The numbers of CD1c+ mDCs did not significantly differ between the groups, although a trend existed for somewhat lower absolute and relative counts in patients (8.58 vs. 10.8 cells/µl, *P* = 0.085; 0.170 vs. 0.193%, *P* = 0.149, Fig. 2A and 2B). The subpopulation of mDCs expressing BDCA3 was, however, reduced in CD (0.348 vs. 0.724 cells/µl, *P* = 0.002; 0.00750 vs. 0.0128%, *P* = 0.004, Fig. 2A and 2B). No difference in the plasma levels of the DC-growth factor FLT3 ligand was seen between the patients and controls (data not shown).

We observed a negative correlation between the endoscopic disease activity (measured by the total SES-CD score) and the relative pDC (ρ = −0.499, *P* = 0.035, Fig. 2C) and BDCA3+ mDC counts (ρ = −0.564, *P* = 0.018, Fig. 2C), and the relative BDCA3+ mDC counts also negatively correlated with C-reactive protein levels (ρ = −0.533, *P = *0.041).

### Expression of DC Surface Markers

**Figure 3:**
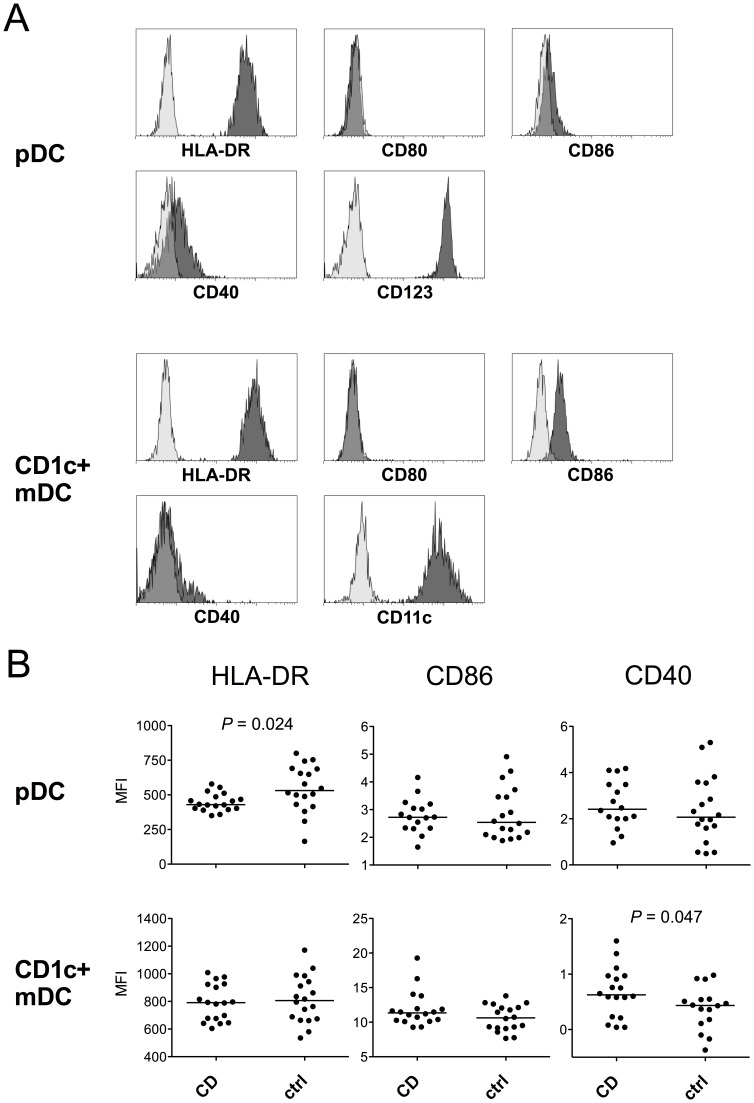
Expression of maturation markers on DCs. pDCs and CD1c+ mDCs were identified as shown in Fig. 1, and the expression of maturation markers was assessed by flow cytometry. The expression of CD123 or CD11c is shown to demonstrate the specificity of DC subtype identification. Light-colored histograms represent isotype controls. A representative example of a healthy control is shown (A). pDCs from CD patients exhibit lower expression of HLA-DR, whereas in CD1c+ mDCs no difference between the groups is observed. Low expression of CD40 is seen on both DC subtypes, the levels on CD1c+ mDCs being, however, higher in CD patients. Values represent median fluorescence intensity (MFI) after subtraction of isotype control fluorescence. Horizontal lines indicate medians for each group (B).

Circulating pDCs from CD patients showed lower levels of HLA-DR expression (median 430 vs. 531 AU, *P* = 0.024, Fig. 3B), whereas no difference was detected for CD86 or CD40. Although the expression of CD40 was low on both DC subtypes (Fig. 3A), somewhat increased levels were observed on CD1c+ mDCs from CD patients (median 0.625 vs. 0.435 AU, *P* = 0.047, Fig. 3B). HLA-DR or CD86 expression on CD1c+ mDCs did not differ between the groups (Fig. 3B). CD80 staining intensity remained at the isotype control level on both DC subtypes (Fig. 3A). Due to the low numbers of BDCA3+ mDCs in the circulation, these cells could only be studied for HLA-DR expression, in which patients did not differ from healthy controls (data not shown). None of the activation/maturation markers correlated with endoscopic disease activity (data not shown).

### IL-6 Levels are Systemically Elevated in CD

**Figure 4:**
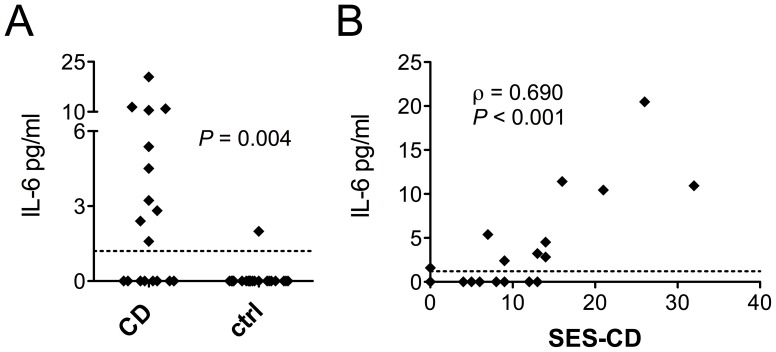
IL-6 is systemically elevated in CD and correlates with the endoscopic disease activity. IL-6 levels in plasma were measured in CD patients and control subjects using a bead-based immunoassay. Measurable quantities (>1.2 pg/ml) of IL-6 were detected in 10 out of 22 CD patients, while IL-6 concentration exceeded the detection limit in only one out of 21 control subjects (A). In CD, levels of IL-6 show a strong positive correlation with the endoscopic disease activity measured by the SES-CD score (B). The dotted line represents the detection limit.

To elucidate the relationship between systemic cytokine levels and the findings in DC analyses, plasma concentrations of selected cytokines were measured. IL-6 was present at detectable concentrations (>1.2 pg/ml) in 10 out of 22 CD patients, whereas it could only be measured in one out of 21 control subjects (*P* = 0.004, Fisher’s exact test, Fig. 4A). IL-6 levels in patients strongly correlated with the disease activity measured by the SES-CD score (ρ = 0.690, *P*<0.001, Fig. 4B). IL-17A was detectable (>13.7 pg/ml) in approximately half of the individuals in both groups (total mean of patients 19.4 pg/ml vs. 23.6 pg/ml in controls), whereas concentrations of IFN-γ, IFN-α, IL-10, and IL-22 were below the limit for a reliable quantitation in most individuals in both groups (data not shown).

### pDCs from CD Patients Show Decreased STAT3 Responses to IL-6 Stimulation

**Figure 5:**
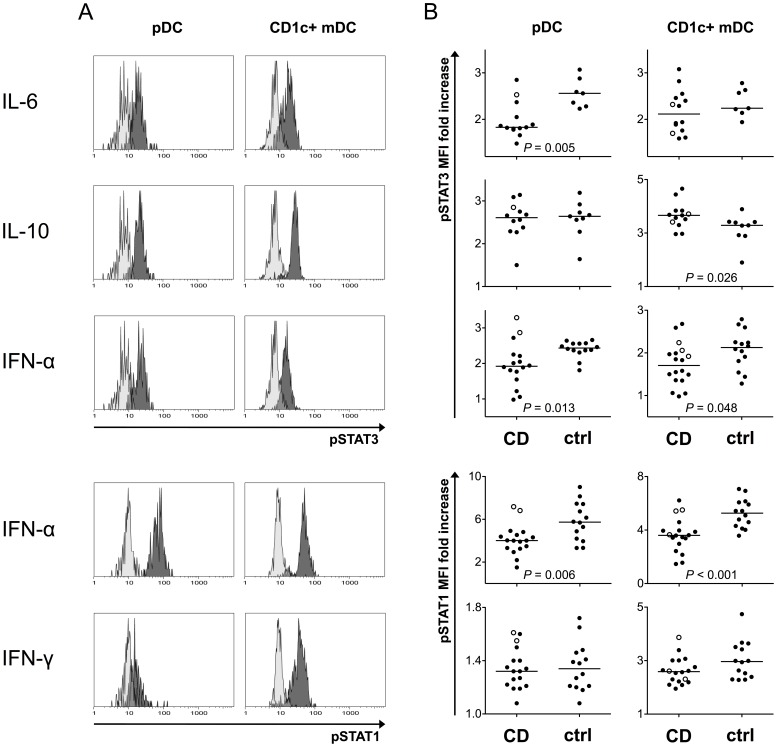
DCs from CD patients exhibit altered levels of phosphorylated STATs after cytokine stimulation. Heparinized whole blood was stimulated with human recombinant cytokines for 10 minutes, and cells were then immediately fixed, permeabilized, and stained for phosphorylated STATs. CD1c+ mDCs and pDCs were identified as shown in Fig. 1B and 1C, respectively. Light-colored histograms represent unstimulated samples. A representative example of pSTAT staining patterns after cytokine stimulation is shown in a healthy control (A). pDCs from patients exhibit a decreased response to IL-6, whereas IL-10 induces similar levels of pSTAT3 in both groups. In CD1c+ mDCs, pSTAT3 levels after IL-10 stimulation are higher in patients, and no difference is seen in the IL-6 response. IFN-α stimulation results in a lower levels of pSTAT1 and pSTAT3 in both DC subsets in CD, but pSTAT1 responses to IFN-γ stimulation are similar with the healthy controls. Values represent fold increase of median fluorescence intensity over unstimulated control. Infliximab-treated patients are indicated by open circles. Horizontal lines indicate median levels (B).

IL-6-induced STAT3 phosphorylation was attenuated in pDCs from CD patients (median MFI fold increase 1.83 vs. 2.56, *P* = 0.005, Fig. 5), whereas no difference was observed in mDCs (1.94 vs. 2.24, NS, Fig. 5). The levels of phosphorylated STAT3 induced by the IL-6 treatment did not correlate with disease activity or plasma IL-6 concentration in either DC subtype in patients (data not shown), which suggests that the attenuated response to IL-6 in pDCs was not due to the elevated systemic IL-6.

**Figure 6:**
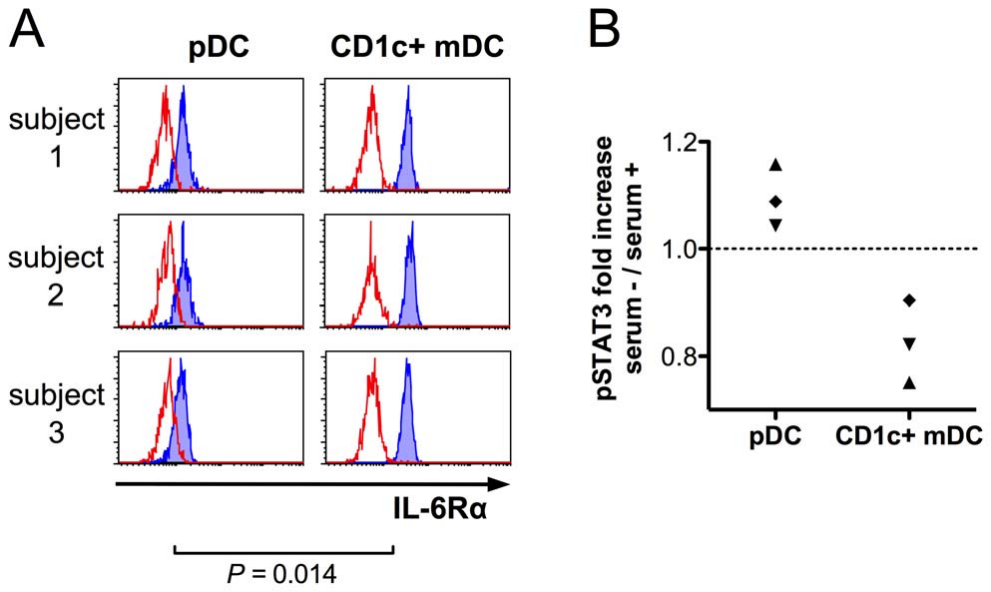
pDCs express cell-surface IL-6Rα and are not dependent on the presence of serum components for IL-6 signal transduction. Flowcytometric analysis of IL-6Rα expression on DC subsets was performed in whole blood samples from healthy volunteers. IL-6Rα is expressed on pDCs, although in lower levels than on CD1c+ mDCs (A). PBMCs were isolated from fresh peripheral blood samples from three healthy volunteers in independent experiments. Cells were incubated at 37°C in RPMI 1640 culture medium (supplemented with 2 mM L-glutamine) either with 1/3 vol. of autologous serum or without serum for one hour prior to stimulation with 50 ng/ml of recombinant IL-6 for 10 minutes (in the presence or absence of serum), followed by flowcytometric DC identification and pSTAT3 measurement using similar gating strategy as indicated in Fig. 1. Despite lower IL-6Rα expression on pDCs, IL-6-induced pSTAT3 response in pDCs is not impaired in the absence of autologous serum, whereas mDCs (serving as intrinsic controls) exhibit somewhat reduced responses when stimulated in serum-free conditions. Samples from each donor are indicated with different symbols (B).

As many cell types lack IL-6 receptor α chain (IL-6Rα) and thus rely on soluble IL-6 receptor (which is present, e.g., in serum) for IL-6 signaling [34], we compared the expression of IL-6Rα between the DC subtypes in healthy donors. The flowcytometric analysis revealed that both mDCs and pDCs expressed IL-6Rα, but the levels were lower on the latter (Fig. 6A). However, IL-6-induced STAT3 signaling in pDCs was not dependent on the presence of serum components (Fig. 6B).

### STAT Responses to IL-10 and IFN-α in DCs are Altered in CD

In pDCs, IL-10 stimulation led to a similar induction of pSTAT3 in both groups (median MFI fold increase 2.61 vs. 2.64, NS, Fig. 5). However, patients’ mDCs responded to IL-10 with a higher level of STAT3 phosphorylation (3.66 vs. 3.29, *P* = 0.026, Fig. 5).

pSTAT1 response to IFN-α stimulation was reduced in pDCs (4.01 vs. 5.74, *P* = 0.006, Fig. 5) and mDCs in CD (3.61 vs. 5.27, P<0.001, Fig. 5), and the IFN-α-induced pSTAT3 level was also decreased in both pDCs (1.92 vs. 2.44, P = 0.013, Fig. 5) and mDCs (1.71 vs. 2.13, *P* = 0.048, Fig. 5). A positive correlation between the endoscopic disease activity (SES-CD score) and INF-α-induced pSTAT3 (ρ = 0.698, *P* = 0.003) in pDCs was seen in CD. In mDCs, the cytokine-induced pSTAT levels did not correlate with the disease activity (data not shown). No association was found between any of the pSTAT responses and disease phenotype (data not shown). pSTAT1 and pSTAT3 intensities in unstimulated samples (after subtraction of isotype control MFI) did not significantly differ between the groups (data not shown).

Since it is possible that CD medication could have influenced STAT phosphorylation, we tested whether the untreated patients differed from healthy individuals. Although the untreated group consisted of only three individuals, the reduced induction of pSTAT3 by IL-6 stimulation, and both pSTAT1 and pSTAT3 by IFN-α stimulation, remained statistically significant in pDCs (*P* = 0.017, *P* = 0.027, and *P* = 0.020, respectively).

### No Transcription of IL-6 or IFN-α could be Detected in Blood-borne pDCs

To assess whether hyporesponsive IL-6- and IFN-α-induced STAT phosphorylation in CD could be related to an autocrine production of the respective cytokines in pDCs, highly purified cells were subjected to RT-qPCR analysis immediately following the isolation. No transcription of either cytokine was detected in any of the pDC samples of patient (n = 11, 73% with active disease, i.e., SES-CD score ≥4) or control subject (n = 11) origin (data not shown).

### IL-6-treated pDCs Promote Th2 Deviation

**Figure 7:**
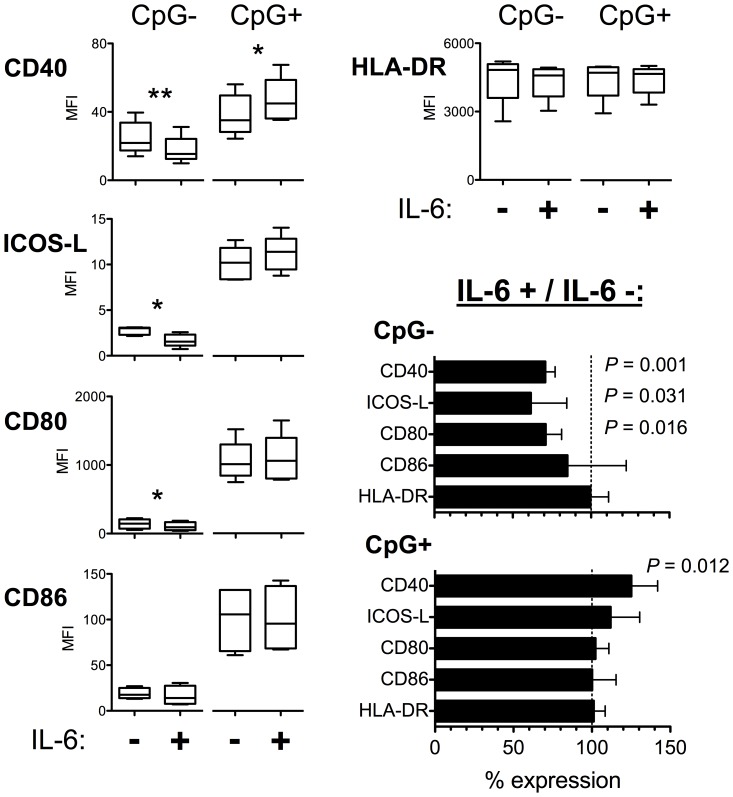
IL-6 modulates pDC maturation. Human pDCs from healthy volunteers were either treated with 50 ng/ml of IL-6 or cultured only in the presence of 10 ng/ml of IL-3 (to maintain pDC viability), with or without stimulation with CpG, for two days. IL-6 inhibits the phenotypic maturation induced by the culture with IL-3. In contrast, IL-6 further upregulates CD40 expression on pDCs when stimulated with CpG-rich hypomethylated oligodeoxynucleotides (mimicking fragments of microbial DNA). Freshly isolated pDCs at d0 consistently had high levels of HLA-DR expression and typically low levels of CD86 and CD40, whereas the staining intensities for CD80 and ICOS-L were at the isotype control level (data not shown). % expression values (in the bottom right part of the figure) were calculated for each experiment ((expression in the presence of IL-6/expression in the absence of IL-6) ×100) and presented as mean ± SD. Flow cytometry data from five independent experiments are presented. * *P*<0.05; ** *P*<0.01.

As patients with CD showed pDC-restricted IL-6 signaling defect, we examined the influence of IL-6 on the activation of pDCs. Using isolated cells from healthy donors, we found that exogenous IL-6 inhibited the phenotypic maturation of pDCs when cultured with pDC-growth factor IL-3 (Fig. 7). In contrast, IL-6 enhanced the upregulation of CD40 expression in the additional presence of CpG in the 2d culture (Fig. 7). No differences were, however, observed in the expression of mRNA transcripts for ICOS ligand, IDO, and programmed cell death 1 ligand 1 (PD-L1) at 16 h (N = 7, data not shown). IL-6 treatment did not affect pDC viability (data not shown).

**Figure 8:**
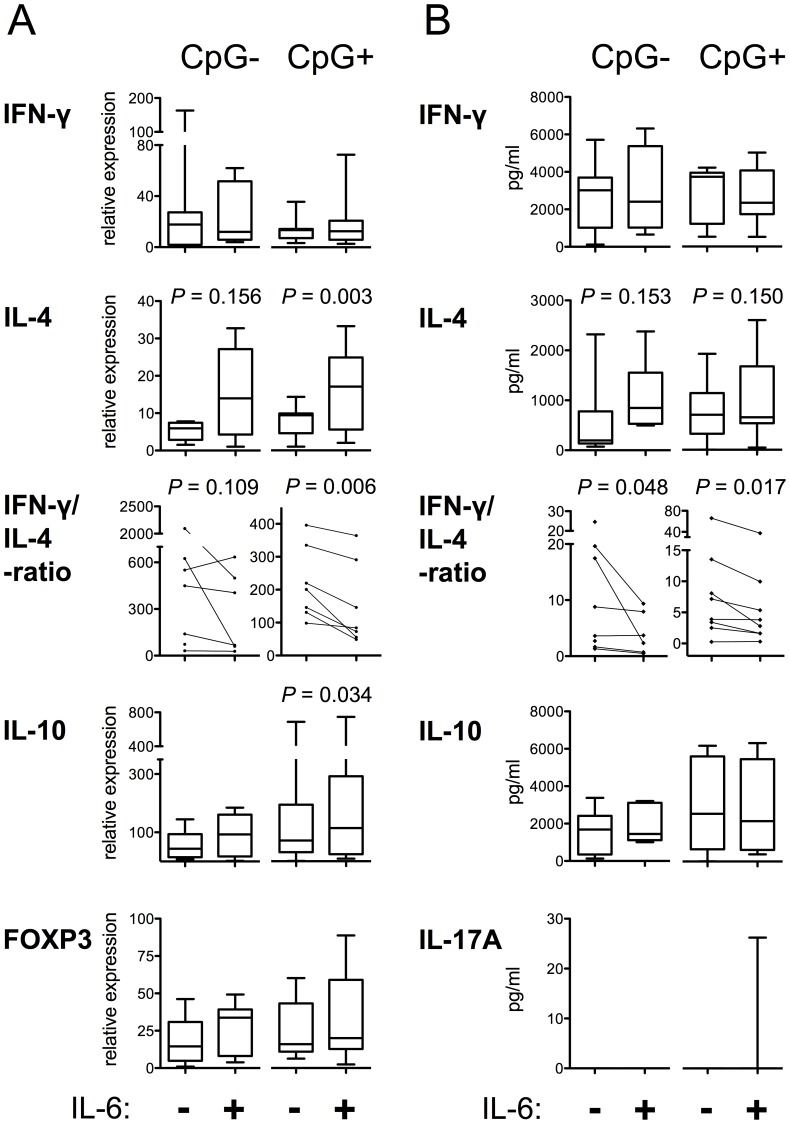
IL-6-treated pDCs promote Th2-type responses. pDCs were matured for two days with IL-3 and the indicated treatments (IL-6 and CpG). Allogeneic naive CD4+ T cells were primed for six days with the pDCs and then restimulated for 16 hours with anti-CD3 and anti-CD28 antibodies. The levels of mRNA transcripts were analyzed by RT-qPCR. Induction of IL-4 is significantly enhanced by the IL-6 treatment of pDCs in the presence of the TLR9 ligand CpG. In addition, the expression of IL-10 mRNA transcripts is higher (A). The concentrations of Th signature cytokines in culture supernatants were determined. IFN-γ to IL-4, i.e., Th1/Th2 ratio, is reduced by the IL-6 treatment of pDCs both in the presence and absence of CpG during the pDC maturation (B).

We next assessed how IL-6-exposed pDCs modulate the cytokine response of T cells. As demonstrated in Fig. 8, IL-6 treatment of pDCs from healthy donors enhanced IL-4 mRNA upregulation in naive allogeneic CD4+ T cells, and a reduced ratio of secreted IFN-γ to IL-4, i.e., Th1/Th2 signature cytokine ratio, was observed irrespective of the presence of CpG during the pDC maturation. In addition, the expression of IL-10 mRNA transcripts was also significantly increased by the IL-6 treatment of CpG-activated pDCs used for priming (Fig. 8A). No differences in IL-10 protein were however detected in culture supernatants (Fig. 8B), which may be explained by the relatively short culture time. pDCs did not induce IL-17A responses from naive T cells in any of our experimental settings (Fig. 8B), and FOXP3 mRNA expression was not affected by the presence of exogenous IL-6 in pDC maturation (Fig. 8A). IL-6 treatment of pDCs had no significant effect on T-cell proliferation (data not shown).

## Discussion

We demonstrate here altered cytokine-induced STAT1 and STAT3 responses of blood-derived DCs in CD. In addition, the numbers of pDCs and BDCA3+ mDCs were decreased.

We observed an attenuated signaling response to IFN-α in both pDCs and CD1c+ mDCs in patients with CD. IFN-α signaling in DCs plays an important role in their activation [35]. In its absence, reduced antigen uptake, lower MHC II expression, and poor CD4+ T cell priming is seen [36]. Decreased HLA-DR expression on pDCs from CD patients was also detected in the present study, in addition to the impaired IFN-α signaling, which could be related to an attenuated or inappropriate function of pDCs by affecting their antigen presentation. Of note, IFN-α signaling in DCs is also critically involved in the cross-priming of cytotoxic T cells [37], important for the antiviral defense, but also for the eradication of intracellular bacteria. In addition, IFN-α is a potent survival factor for pDCs [38].

It was recently reported that DC-specific knockout of STAT3 (in Stat3^flox/flox^ × CD11cCre mice), which affects both mDCs and pDCs, leads to a lifelong ileocolitis resembling CD [29], identifying the deficient STAT3 activity in DCs as an important factor in the development of mucosal inflammation. In this model, resistance to IL-10-mediated suppression was seen in STAT3-deficient DCs, whereas the role of IL-6, another cytokine with STAT3-dependent signal transduction, was not dissected in DC regulation. In our series of CD patients, not selected according to the STAT3 genotype, we observed deficient IL-6/STAT3 signaling in pDCs, while DCs’ responsiveness to IL-10 was intact or even enhanced. Thus, our results extend the concept of deficient STAT3 activity in DCs to human CD and implicate IL-6 and pDCs.

Our observation of increased plasma IL-6 levels in CD patients is in agreement with previous reports [39], [40]. The elevated systemic IL-6 could be a feasible explanation for the impaired IL-6 response we observed in pDCs. However, IL-6-induced STAT3 phosphorylation was not considerably affected in mDCs, and plasma IL-6 levels did not correlate with the responses to exogenous IL-6 in either DC subtype. Moreover, we did not find signs of enhanced *ex vivo* production of IL-6 or IFN-α in pDCs isolated from CD patients, even though increased IL-6 production from *in vitro* cultured pDCs has been reported in CD [41]. Accordingly, our findings do not support the view of impaired IL-6 and IFN-α signaling being due to negative feedback mechanisms induced by an autocrine secretion of these cytokines.

Increased maturation of liver pDCs has been demonstrated in IL-6 knockout mice [42], but the role of IL-6 in pDC regulation has not, to our knowledge, been previously directly assessed with human cells. We found that exogenous IL-6 inhibited the phenotypic maturation of healthy donor pDCs during *in vitro* culture. In the presence of CpG, however, an increased CD40 induction was instead seen in IL-6-treated pDCs, so no straightforward conclusions can be drawn. IL-6-treated pDCs also induced a higher level of IL-4 production from *in vitro* differentiated T cells, which was accompanied by a decreased IFN-γ to IL-4, i.e., Th1/Th2 signature cytokine ratio, and by an increased expression of IL-10 transcripts. Notwithstanding this relatively modest effect on T cells, we hypothesize that the attenuated IL-6 signaling in CD patients’ pDCs may be of significance, given that IFN-γ expression in MLN CD4+ T cells is enhanced in CD [14] and chronic enterocolitis is well-established to develop in IL-10-deficient mice [43]. Even though not enough cells could be obtained for these protocols from patients, these experiments demonstrate a regulatory function for IL-6 on human pDCs.

We observed decreased numbers of pDCs in CD, as reported previously [15]. Data from a number of studies point towards pDCs’ tolerogenic potential [8]–[13]. We found that pDC-primed CD4+ T cells in all experimental conditions produced high quantities of IL-10 upon restimulation, which may signify importance for the reduced pDC numbers in regard to peripheral T cell tolerance in CD. There was also a significant reduction in the minor BDCA3+ mDC population in patients’ circulation. The relative numbers of pDCs and BDCA3+ mDCs in the present study inversely correlated with the disease activity, which is in line with an earlier report [15]. Accordingly, it is also possible that their scarcity in the peripheral circulation is related to migration to the inflamed intestine or to the MLNs [41]. It is of interest whether the decrease in the peripheral blood pDCs or BDCA3+ mDCs could predict disease activation.

CD1c+ mDCs from CD patients exhibited a more activated immunophenotype in terms of CD40 expression, as reported for *in vitro* cultured CD1c+ mDCs [20], and also for intestinal mDCs isolated from lamina propria of CD patients [16], [20]. This could contribute to the pathogenesis of CD by increasing the likelihood of inappropriate T-cell activation. The present study demonstrates that the IL-10 signal, which potently inhibits human DC maturation [44], is transduced more efficiently in CD patients’ mDCs, possibly reflecting a compensatory mechanism.

We realize the limitations in the studies of blood-derived DCs instead of intestinal cells, but there are also problems related to the study of the latter. Most importantly, the influence of bystander mechanisms on STAT phosphorylation would likely be more pronounced and heavily dependent on the degree of inflammation in a given biopsy sample. In the intestinal T cells from CD patients, constitutively enhanced STAT3 phosphorylation has been described [21], [23], which could reflect the local environment, and at least in part, be attributable to increased IL-6 production in the gut/GALT. A recent study demonstrated that intestinal epithelial cell-specific STAT3 deficiency results in altered STAT3 activation in the immune cells and the expansion of IL-17A-secreting intestinal T cells in a colitis model [45]. Accordingly, STAT3 activation in intestinal epithelial cells and T cells, and evidently also in DCs, forms a complex interaction. These studies emphasize the understanding of the cell-cell interaction in the tissue, but also the importance of cytokine-specific and target-cell specific dissection of the STAT3 pathway. STAT3 activation displays cell-specific differences in function. Removal of STAT3-mediated suppression in myeloid cells leads to enterocolitis [46]. In contrast, STAT3 activity in T cells is critically involved in the differentiation of the phenotype that produces IL-17A/F [47], cytokines clearly implicated in CD [48], [49], and increased IL-6-induced STAT3 phosphorylation in peripheral blood T cells and granulocytes has been reported [50] in children with CD-associated genetic variant of *STAT3* [27]. Inflammation-related bystander mechanisms cannot be excluded based on our data, either, although most findings of altered signaling were not associated with disease activity. Moreover, the IFN-α-induced pSTAT3 levels in pDCs, which we found to be decreased in CD, actually showed a positive correlation with the disease activity.

Most patients in our study were receiving medication at the time of analysis - this could not be circumvented due to a low number of untreated patients available. Because pDCs and BDCA3+ mDCs were decreased also as a ratio to total leukocytes, our findings of reduced DC counts are, however, likely not due to a drug-mediated bone marrow suppression. Furthermore, after exclusion of all patients receiving any treatment for CD, the results on IL-6- and IFN-α-induced STAT responses in pDCs remained similar.

To our knowledge, this work represents the first demonstration of an abnormal DC-related STAT signaling in human CD, characterized by the attenuated IL-6/STAT3 axis in pDCs and decreased IFN-α-induced signaling in both DC subtypes. These findings provide evidence for innate immunity alterations that could be connected to the impaired immunoregulation in CD. We also believe that the methodology for the evaluation of cell signaling-related phosphoproteins, optimized by us for primary human DCs, may be of utility in other inflammatory and autoimmune conditions.

## Supporting Information

Figure S1 **Mainly T cells are found within the viable cell fraction after six days of co-culture of pDCs plus CD4+ naive T cells.** pDCs were obtained by negative magnetic selection from blood donor buffy coats, cultured for two to three days in the presence of IL-3, with or without CpG, washed, and used for the induction of activation and proliferation of allogeneic naive T cells (isolated either from blood donor buffy coat or from a regular heparinized venous blood sample) for six days, as described in Materials and Methods. Cells were then stained for flowcytometric analysis with antibodies against pDC and T-cell markers and with 7-AAD viability dye (BDCA2 expression was not assessed, because it is known to be downregulated in culture). Low relative numbers of viable pDCs (CD45^+^7-AAD^-^CD3^-^, CD123^+^/BDCA4^+^/HLA-DR^high^) were observed after six days of co-culture of pDCs and CD4+ naive T cells, and thus no pDC depletion was performed before T-cell restimulation when analyzing T-cell cytokine production. Proliferating T cells (within the CD3+ events) seem to exhibit variable CD123 (IL-3Rα) and HLA-DR staining. BDCA4 intensity in T cells is at the level of isotype control (not shown). Values in graphs indicate the percentage of events in the corresponding gate. Data from two independent experiments are presented.
(TIFF)

Table S1 **Antibodies in flow cytometry.**
(DOCX)
