# Supplementary figures and images for: Dendritic Cells from Crohn’s Disease Patients Show Aberrant STAT1 and STAT3 Signaling
Source: PLoS One. 2013 Aug 7;8(8):e70738. doi: 10.1371/journal.pone.0070738 (PMC3737363; doi:10.1371/journal.pone.0070738)

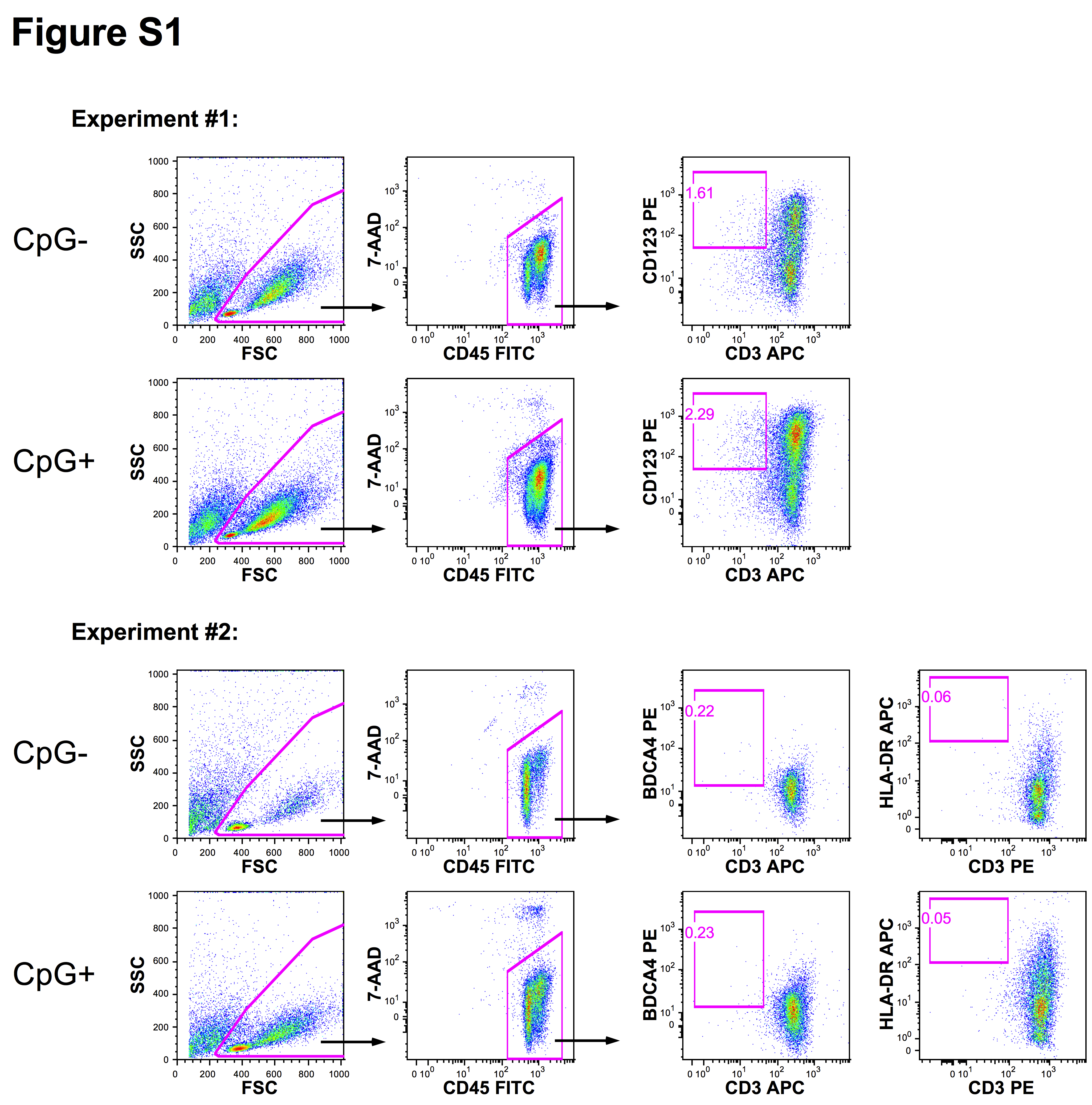

Supplement: Figure S1 — Mainly T cells are found within the viable cell fraction after six days of co-culture of pDCs plus CD4+ naive T cells. pDCs were obtained by negative magnetic selection from blood donor buffy coats, cultured for two to three days in the presence of IL-3, with or without CpG, washed, and used for the induction of activation and proliferation of allogeneic naive T cells (isolated either from blood donor buffy coat or from a regular heparinized venous blood sample) for six days, as described in Materials and Methods. Cells were then stained for flowcytometric analysis with antibodies against pDC and T-cell markers and with 7-AAD viability dye (BDCA2 expression was not assessed, because it is known to be downregulated in culture). Low relative numbers of viable pDCs (CD45+7-AAD-CD3-, CD123+/BDCA4+/HLA-DRhigh) were observed after six days of co-culture of pDCs and CD4+ naive T cells, and thus no pDC depletion was performed before T-cell restimulation when analyzing T-cell cytokine production. Proliferating T cells (within the CD3+ events) seem to exhibit variable CD123 (IL-3Rα) and HLA-DR staining. BDCA4 intensity in T cells is at the level of isotype control (not shown). Values in graphs indicate the percentage of events in the corresponding gate. Data from two independent experiments are presented. (TIFF) [file pone.0070738.s001.tiff]
