# Supplementary material for: Dendritic Cells from Crohn’s Disease Patients Show Aberrant STAT1 and STAT3 Signaling
Source: PLoS One. 2013 Aug 7;8(8):e70738. doi: 10.1371/journal.pone.0070738 (PMC3737363; doi:10.1371/journal.pone.0070738)
Supplement: Table S1 — Antibodies in flow cytometry. (DOCX) [file pone.0070738.s002.docx]

**Table S1. Antibodies in flow cytometry.**

| **Ag** | **Clone** | **Conjugate** | **Manufacturer** |
| --- | --- | --- | --- |
| HLA-DR | L243 | FITC | BD Biosciences |
| CD86 | 2331 | FITC | BD Biosciences |
| CD80 | L307.4 | FITC | BD Biosciences |
| CD45 | 2D1 | FITC | BD Biosciences |
| CD45RA | L48 | FITC | BD Biosciences |
| CD40 | 5C3 | FITC | BD Biosciences |
| pSTAT1 | 4a | A488 | BD Biosciences |
| pSTAT3 | 4/P-STAT3 | A488 | BD Biosciences |
| CD3 | SK7 | PE | BD Biosciences |
| CD123 | 9F5 | PE | BD Biosciences |
| CD80 | L307.4 | PE | BD Biosciences |
| CD40 | 5C3 | PE | BD Biosciences |
| CD11c | S-HCL-3 | PE | BD Biosciences |
| CD4 | SK3 | PE | BD Biosciences |
| ICOS-L | 2D3/B7-H2 | PE | BD Biosciences |
| CD45 | 2D1 | PerCP | BD Biosciences |
| CD3 | SK7 | APC | BD Biosciences |
| CD45RO | UCHL1 | APC | BD Biosciences |
| HLA-DR | G46-6 | APC | BD Biosciences |
| CD14* | TüK4 | PE-Cy5.5 | Caltag/Invitrogen |
| CD19 | SJ25-C1 | PE-Cy5.5 | Caltag/Invitrogen |
| BDCA4 | AD5-17F6 | PE | Miltenyi Biotec |
| CD1c | AD5-8E7 | APC | Miltenyi Biotec |
| BDCA2 | AC144 | APC | Miltenyi Biotec |
| BDCA3 | AD5-14H12 | APC | Miltenyi Biotec |
| IL-6Rα | 17506 | CFL | R&D Systems |
| mouse IgG1 ctrl | X40 | FITC | BD Biosciences |
| mouse IgG2a ctrl | X39 | FITC | BD Biosciences |
| mouse IgG2a ctrl | MOPC-173 | A488 | BD Biosciences |
| mouse IgG1 ctrl | X40 | PE | BD Biosciences |
| mouse IgG2a ctrl | X39 | PE | BD Biosciences |
| mouse IgG2b ctrl | 27-35 | PE | BD Biosciences |
| mouse IgG1 ctrl | X40 | APC | BD Biosciences |
| mouse IgG1 ctrl | MOPC-21 | PE-Cy5.5 | Caltag/Invitrogen |
| mouse IgG2a ctrl | 5.205 | PE-Cy5.5 | Caltag/Invitrogen |
| mouse IgG2a ctrl | S43.10 | APC | Miltenyi Biotec |
| mouse IgG2b ctrl | cat# SFL691PE | PE | Serotec |
| mouse IgG1 ctrl | 11711 | CFL | R&D Systems |

Abbreviations: FITC = fluorescein isothiocyanate; A488 = AlexaFluor 488; PE = phycoerythrin; PerCP = peridinin chlorophyll protein; APC = allophycocyanin, PE-Cy5.5 = phycoerythrin-Cy5.5; CFL = carboxyfluorescein. In case clone is not specified by the manufacturer, the catalogue number is shown instead. *The fluorescence intensity of the conjugate was kept within an acceptable range by adding purified antibody of the same clone as a ‘cold competitor’ at a ratio of 2:1 (purified to conjugated) prior to use. The optimal concentrations for antibodies were determined by titration experiments.
